# Irbesartan Ameliorates Diabetic Nephropathy by Suppressing the RANKL-RANK-NF-*κ*B Pathway in Type 2 Diabetic db/db Mice

**DOI:** 10.1155/2016/1405924

**Published:** 2016-01-06

**Authors:** Xiao-Wen Chen, Xiao-Yan Du, Yu-Xian Wang, Jian-Cheng Wang, Wen-Ting Liu, Wen-Jing Chen, Hong-Yu Li, Fen-Fen Peng, Zhao-Zhong Xu, Hong-Xin Niu, Hai-Bo Long

**Affiliations:** ^1^Department of Nephrology, ZhuJiang Hospital, Southern Medical University, Guangzhou 510280, China; ^2^Department of Gerontology, ZhuJiang Hospital, Southern Medical University, Guangzhou 510280, China; ^3^Department of Emergency, ZhuJiang Hospital, Southern Medical University, Guangzhou 510280, China

## Abstract

The receptor activator of NF-*κ*B ligand (RANKL) and its receptor RANK are overexpressed in focal segmental glomerular sclerosis (FSGS), IgA nephropathy (IgAN), and membranous nephropathy (MN). However, the expression and the potential roles of RANKL and RANK in diabetic nephropathy (DN) remain unclear. Irbesartan (Irb) has beneficial effects against diabetes-induced renal damage, but its mechanisms are poorly understood. Our present study investigated the effects of Irb in DN and whether the renal protective effects of Irb are mediated by RANKL/RANK and the downstream NF-*κ*B pathway in db/db mice. Our results showed that db/db mice revealed severe metabolic abnormalities, renal dysfunction, podocyte injury, and increased MCP-1; these symptoms were reversed by Irb. At the molecular level, RANKL and RANK were overexpressed in the kidneys of db/db mice and Irb downregulated RANKL and RANK and inhibited the downstream NF-*κ*B pathway. Our study suggests that Irb can ameliorate DN by suppressing the RANKL-RANK-NF-*κ*B pathway.

## 1. Introduction

DN, which is characterized by progressive albuminuria and a gradual decline in glomerular filtration rate, is a distinct kidney disease that is becoming the single most important cause of end-stage renal disease (ESRD) throughout the world [1, 2]. Approximately 30% of patients with type 1 diabetes mellitus develop DN and approximately 25 to 30% of patients with type 2 diabetes mellitus (T2DM) will develop overt DN [3, 4]. Diverse pathogenic mechanisms contribute to DN, including advanced glycation end products, activation of protein kinase C, and overexpression of different growth factors. However, accumulating studies suggest that podocyte injury [5, 6] and inflammatory processes [7–9] also play vital roles in the development and progression of DN. Podocyte injury has been observed in the kidneys of diabetic patients and animal models and can precede and predict the occurrence of proteinuria [5, 6]. Additionally, MCP-1, which is the strongest known chemotactic factor for monocytes, is a key inflammatory factor in the pathogenesis and progression of DN [7, 9, 10]. Thus, therapies that protect podocytes from injury and control inflammation are very important to the treatment of DN.

Angiotensin II receptor blocker (ARB) offers effective renoprotection in diabetic animal models and diabetic patients. In animal studies, Irb significantly lowers the blood lipid level and improves the kidney function of diabetic mice and rats [11, 12]. In clinical trials, Irb and telmisartan are effective in reducing proteinuria in patients with DN [13, 14]. Additionally, Irb increases glomerular synaptopodin immunoreactivity, a marker of functional podocytes, and exerts a beneficial effect on diabetes-induced renal inflammation [15]. However, the molecular mechanism through which ARB reverses kidney dysfunction remains incompletely understood.

The RANKL system consists of a triad of proteins: type II membrane protein RANKL, its biological receptor RANK, and decoy receptor osteoprotegerin (OPG). RANKL signaling has been extensively studied in osteoclasts. Binding of RANK initiates a signaling cascade that activates NF-*κ*B and mitogen-activated protein kinases, which induce osteoclastogenesis and lead to osteoclast-related diseases such as osteoporosis [16, 17]. Recently, Liu et al. [18] identified a new role of RANKL and RANK. They reported that RANKL and RANK were overexpressed in FSGS, IgAN, and MN and were specifically expressed in podocytes. A genome-wide association study also revealed a polymorphism in the podocyte receptor RANK that leads to a decline in renal function in coronary patients [19]. Kiechl et al. found that, as the chief upstreaming molecules of NF-*κ*B, RANKL and RANK play a pivotal role in the pathophysiology of T2DM [20]. However, little is known about the potential role of RANKL and RANK in the development of DN.

The present study investigated the role of RANKL and RANK in type 2 DN and whether the renoprotective effects of Irb were mediated by the RANKL-RANK-NF-*κ*B pathway.

## 2. Materials and Methods

### 2.1. Animal Model and Drug Treatment

Male db/db mice in a C57BL/KsJ (BKS.Cg-Dock7m +/+ Leprdb/J) background and their age-matched, normal, wild-type db/m littermates were obtained from the Animal Model Research Center of Nanjing University. Mice were housed in the Laboratory Animal Center of Sun Yat-sen University under controlled temperature (20–22°C), light (alternating 12-hour light/dark cycle), and humidity (50%–60%) conditions and received food and water freely. After 1 week of acclimatization, the db/db mice were randomly divided into 2 groups (*n* = 12 each): db/db mice and db/db mice+Irb (50 mg/kg/day). Additionally, 12 db/m mice served as the control group. After 12 weeks of Irb treatment, the mice were 20 weeks old and were sacrificed for this experiment. The Animal Care and Use Committee of Sun Yat-sen University reviewed and approved all animal studies and all procedures were in compliance with the National Institutes of Health Guide for Care and Use of Laboratory Animals (Publication number 85-23, revised 1985).

### 2.2. Serum Measurements

The mice were fasted overnight and then blood was sampled from the eyes of the mice. Serum triglycerides (TG), total cholesterol (TC), blood urea nitrogen (BUN), and serum creatinine (Scr) levels were determined using an automatic analyzer (Roche, Basel, Switzerland). Fasting blood glucose (FBG) was measured using a glucose meter (Roche Diagnostics GmbH, Mannheim, Germany). Fasting insulin (FINS) was measured by an ELISA assay (Jiancheng, Nanjing, China).

### 2.3. Urinary Albumin to Creatinine Ratio (ACR) Assay

Mice were placed in metabolic cages for urine collection at 8, 12, 16, and 20 weeks of age. The collected urine specimens were centrifuged at 10,000 ×g for 5 minutes at 4°C. The microalbumin level in the urine supernatants was measured using a murine microalbuminuria ELISA kit (Exocell, Philadelphia, PA, USA), and the creatinine in the urine was assayed by the Creatinine Companion kit (Exocell, Philadelphia, PA, USA). Then, the ACR was calculated as previously described [21].

### 2.4. Histological Analysis

Kidneys were cut longitudinally, and the kidney cortexes were fixed with neutral formalin for performing paraffin sectioning. The paraffin blocks were cut into 3 *μ*m sections, stained with Periodic Acid-Schiff (PAS), and used for mesangial analyses. Thirty glomeruli per animal were analyzed with a 20x objective lens by two independent, masked investigators. Mesangial expansion was evaluated using a semiquantitative scoring system as follows [22]: 0, no expansion; 1, expansion less than 25%; 2, expansion between 25% and 50%; 3, expansion between 50% and 75%; and 4, expansion greater than 75% of the mesangial area.

### 2.5. Transmission Electron Microscopy (TEM)

Kidney cortical tissue from each group was fixed with glutaraldehyde, embedded in Epon resin, and thinly sliced (60 to 100 nm) using an ultramicrotome (Leica). Then, the ultrathin sections were collected on copper grids and stained with uranyl acetate and lead citrate. Finally, transmission electron microscopy was performed as previously described [23].

### 2.6. Immunohistochemistry (IHC)

Murine renal tissue from each group was fixed for immunohistochemical staining in 10% neutral formalin, processed in the standard manner, and cut into 3 *μ*m sections. Slides were deparaffinized, hydrated in ethyl alcohol, and washed in tap water. Then, the slides underwent antigen retrieval and blocking with 5% BSA. To assess RANKL, RANK, and nephrin in renal tissues, the sections were incubated with an anti-RANKL polyclonal antibody (RANKL, 1 : 50, Santa Cruz, CA, USA), an anti-RANK polyclonal antibody (RANK, 1 : 50, Santa Cruz, CA, USA), or an anti-nephrin polyclonal antibody (nephrin, 1 : 50, Santa Cruz, CA, USA) overnight at 4°C. After washing, a secondary donkey anti-rabbit antibody was added for 30 minutes (min) at 37°C; the slides were then washed and incubated with DAB for 2 min before counterstaining with hematoxylin. Sections were viewed and imaged with a light microscope (Ni-U, Nikon Corporation, Tokyo, Japan). Images were analyzed quantitatively by Image-Pro Plus 6.0 (IPP, Media Cybernetics, Inc., USA).

### 2.7. Enzyme-Linked Immunosorbent Assay

At the end of the experiment, urine samples from the mice were collected and centrifuged at 10,000 ×g for 5 min at 4°C to remove the debris from the supernatants. Then, the supernatants were assayed using a solid-phase quantitative sandwich ELISA kit for MCP-1, as previously described [24]. The urinary MCP-1 concentration was normalized to the urinary creatinine concentration.

### 2.8. Quantitative Real-Time PCR Analysis

RNA from the kidneys of each group of mice was extracted as previously described [21]. The primers for qPCR were as follows: MCP-1 forward: 5′-CCC AAT GAG TAG GCT GGA GA-3′, reverse: 5′-TCT GGA CCC ATT CCT TCT TG-3′, and GAPDH forward: 5′-ATT GTC AGC AAT GCA TCC TG-3′, reverse: 5′-ATG GAC TGT GGT CAT GAG CC-3′. All data were normalized to GAPDH, and the expression levels were analyzed by the 2^−DDCT^ method.

### 2.9. Western Blot Analysis

Total proteins were extracted from the kidneys, separated on 10% SDS-polyacrylamide gels, and transferred onto PVDF membranes (Millipore, Bedford, MA). The membrane was blocked for 1 h with Tris-buffered saline (20 mM Tris-HCl, 140 mM NaCl, pH 7.6) containing 5% nonfat dry milk, washed with TBS containing 0.1% Tween-20, and incubated overnight at 4°C with a primary antibody. The primary RANKL rabbit mAb and RANK rabbit mAb were purchased from Santa Cruz Biotechnology (Santa Cruz, CA, USA). NF-*κ*B p65 rabbit mAb, p-NF-*κ*B p65 rabbit mAb, p-I*κ*B*α* rabbit mAb, and I*κ*B*α* mouse mAb were purchased from Cell Signaling Technology (Danvers, MA). *β*-actin mouse mAb and HRP-conjugated secondary antibodies were purchased from EarthOx (EarthOx, LLC, San Francisco, CA, USA). The protein band intensities were quantified with Quantity One 4.6.2 analysis software (Quantity One, Bio-Rad Laboratories, Inc., USA), which was provided with the Kodak 2000MM System (Eastman Kodak Company, Rochester, New York, USA).

### 2.10. Statistical Analysis

The results are presented as the mean ± S.D. Differences among multiple groups were analyzed by one-way ANOVA followed by a *t*-test to detect between-group differences. A two-sided *P* < 0.05 was considered significant. All analyses were performed using IBM SPSS Statistics 20.0 (SPSS, Chicago, IL, USA).

## 3. Results

### 3.1. Effect of Irb on Metabolic Indices

At the end of the experiment, FBG, FINS, TC, TG, and body weight (BW) levels were significantly increased in the db/db mice compared with the db/m mice (Table 1). After 12 weeks of treatment with Irb, the TC and TG levels of the db/db mice decreased from 5.21 ± 0.72 mmol/L and 2.55 ± 0.60 mmol/L to 3.12 ± 0.93 mmol/L and 1.08 ± 0.61 mmol/L, respectively. However, there were no significant differences in the FBG, FINS, and BW levels between db/db mice treated with and without Irb.

### 3.2. Irb Alleviated Diabetes-Induced Renal Dysfunction

**Figure 1:**
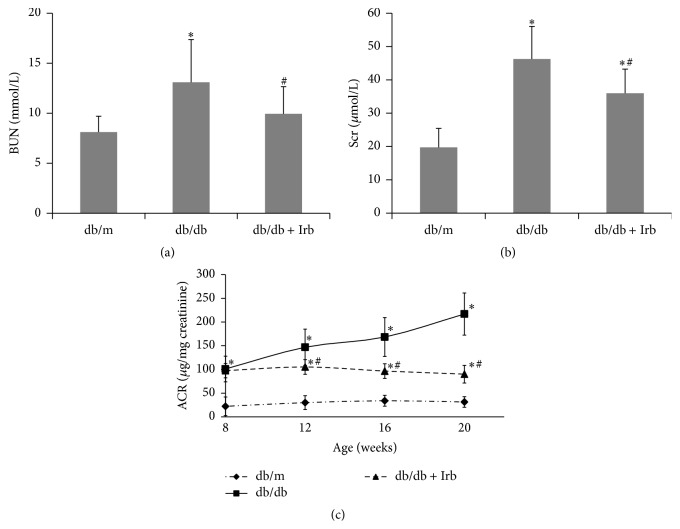
Irb alleviated diabetes-induced renal dysfunction. (a) BUN was increased in db/db mice and reversed significantly by Irb. (b) Scr was increased in db/db mice and reversed significantly by Irb. (c) ACR increased dramatically in db/db mice in an age-dependent method, but this increase was reversed significantly by Irb. The bars in panel (a–c) show the mean expression level in arbitrary units (error bars, S.D.). ^*∗*^ *P* < 0.05 compared with db/m; ^#^ *P* < 0.05 compared with db/db, *t*-test.

Blood urea nitrogen (BUN), serum creatinine (Scr), and urinary albumin to creatinine ratio (ACR) were evaluated to assess the renal function of the mice. Both BUN and Scr were measured at the end of the experiment. ACR was detected at 8, 12, 16, and 20 weeks of age. BUN, Scr, and ACR were significantly increased in the db/db mice compared with the db/m mice (Figures 1(a)–1(c)). With age, ACR was increased dramatically in db/db mice but remained stable in db/m mice (Figure 1(c)). After 12 weeks of treatment with Irb, BUN and Scr were significantly decreased compared with the untreated db/db mice (Figures 1(a) and 1(b)). Moreover, Irb markedly slowed the sharp ACR increase (Figure 1(c)). These results indicate that Irb can improve the renal function of db/db mice.

### 3.3. Irb Alleviated Diabetes-Induced Renal Pathological Changes

**Figure 2:**
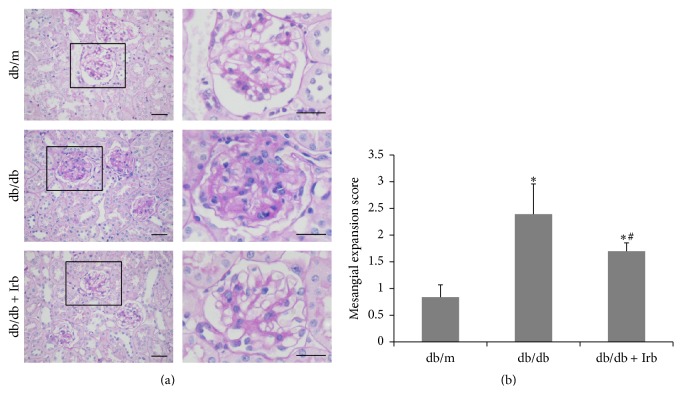
Irb alleviated diabetes-induced renal pathological changes. (a) Representative fields of the glomerulus stained with PAS (scale bars: 50 *μ*m). (b) The mesangial expression score increased in db/db mice and decreased in db/db mice with Irb treatment. The bars in panel (b) show the mean expression in arbitrary units (error bars, S.D.). ^*∗*^ *P* < 0.05 compared with db/m; ^#^ *P* < 0.05 compared with db/db, *t*-test.

At the end of the experiment, kidney tissues were collected for the PAS stain. The db/db mice showed hyaline degeneration in the glomerular afferent arteries and mesangial expansion with major accumulation of mesangial matrix in the glomerulus (Figure 2(a)). The mesangial expansion score was significantly higher in db/db mice than in db/m mice, and this increase was reversed by Irb (Figure 2(b)).

### 3.4. Irb Alleviated Diabetes-Induced Podocyte Injury and Thickening of the GBM

**Figure 3:**
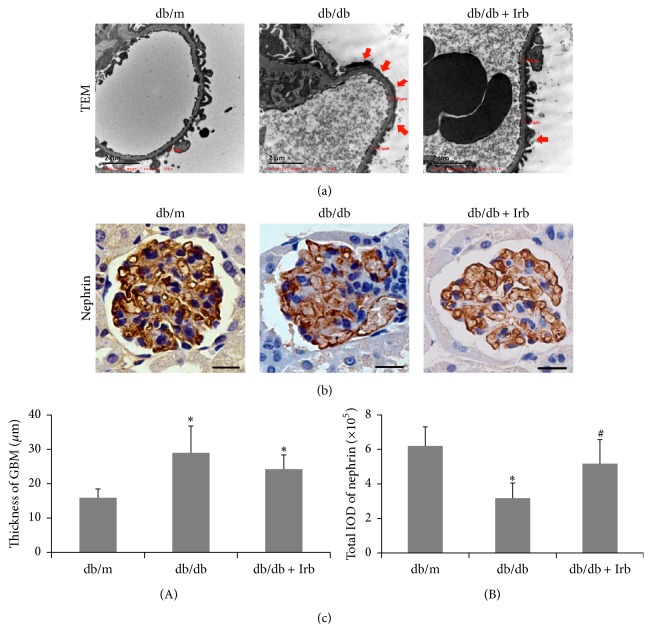
Irb alleviated diabetes-induced podocyte injury and the thickening of the GBM. (a) Representative fields of podocyte foot processes under TEM (scale bars: 2 *μ*m, red arrow indicates podocyte foot process effacement). (b) Representative fields of nephrin, as labeled by immunohistochemical staining (scale bars: 50 *μ*m). (c) Quantification of the GBM thickness (A) and immunohistochemical staining (B). The bars in panel (c) show the mean expression in arbitrary units (error bars, S.D.). ^*∗*^ *P* < 0.05 compared with db/m; ^#^ *P* < 0.05 compared with db/db, *t*-test.

To examine podocyte injury and the thickness of the GBM, kidney tissues were observed via TEM. Using electron microscopy, we noted that in db/db mice, foot process effacement occurred in most of the podocytes, and the thickness of the GBM significantly increased compared with the db/m mice (Figures 3(a) and 3(c)(A)). Moreover, nephrin, a marker of functional podocytes, was detected in kidney tissues by IHC. The results in Figure 3 show discontinuous punctiform or linear expression of nephrin in db/db mice but continuous linear expression of nephrin in db/m mice (Figure 3(b)). Compared with the db/m mice, nephrin was reduced significantly in db/db mice (Figures 3(b) and 3(c)(B)). After 12 weeks of treatment with Irb, the podocyte injury was improved markedly; however, the thickness of the GBM did not change significantly between the db/db mice treated with and without Irb (Figures 3(a)–3(c)).

### 3.5. Irb Attenuated Diabetes-Induced MCP-1 Expression

**Figure 4:**
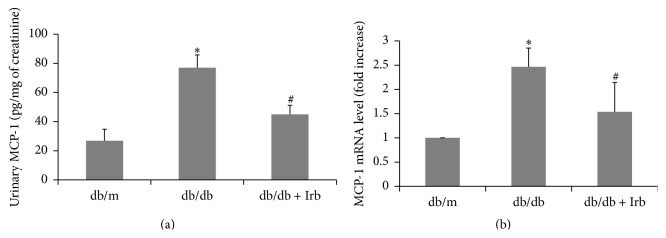
Irb attenuated diabetes-induced MCP-1 expression. (a) Urinary MCP-1 was significantly increased in db/db mice and significantly reduced in db/db mice with treatment of Irb. (b) The mRNA level of MCP-1 in the kidney was increased in the db/db mice and reversed by Irb treatment significantly. The bars in panels (a) and (b) show the mean expression in arbitrary units (error bars, S.D.). ^*∗*^ *P* < 0.05 compared with db/m; ^#^ *P* < 0.05 compared with db/db, *t*-test.

MCP-1 attracts macrophages and promotes inflammation in DN [7, 9, 10]. Thus, we examined the protein level of MCP-1 in the urine of mice and the mRNA level of MCP-1 in kidney tissues. As shown in Figure 4, the urinary concentrations of MCP-1 were significantly increased in db/db mice compared with db/m mice. After Irb treatment, the MCP-1 concentrations were drastically lowered in the urine (Figure 4(a)). Similarly, the MCP-1 mRNA level was also increased in the kidney of db/db mice and was reduced by Irb treatment (Figure 4(b)).

### 3.6. RANKL and RANK Were Overexpressed in db/db Mice and Downregulated by Irb

**Figure 5:**
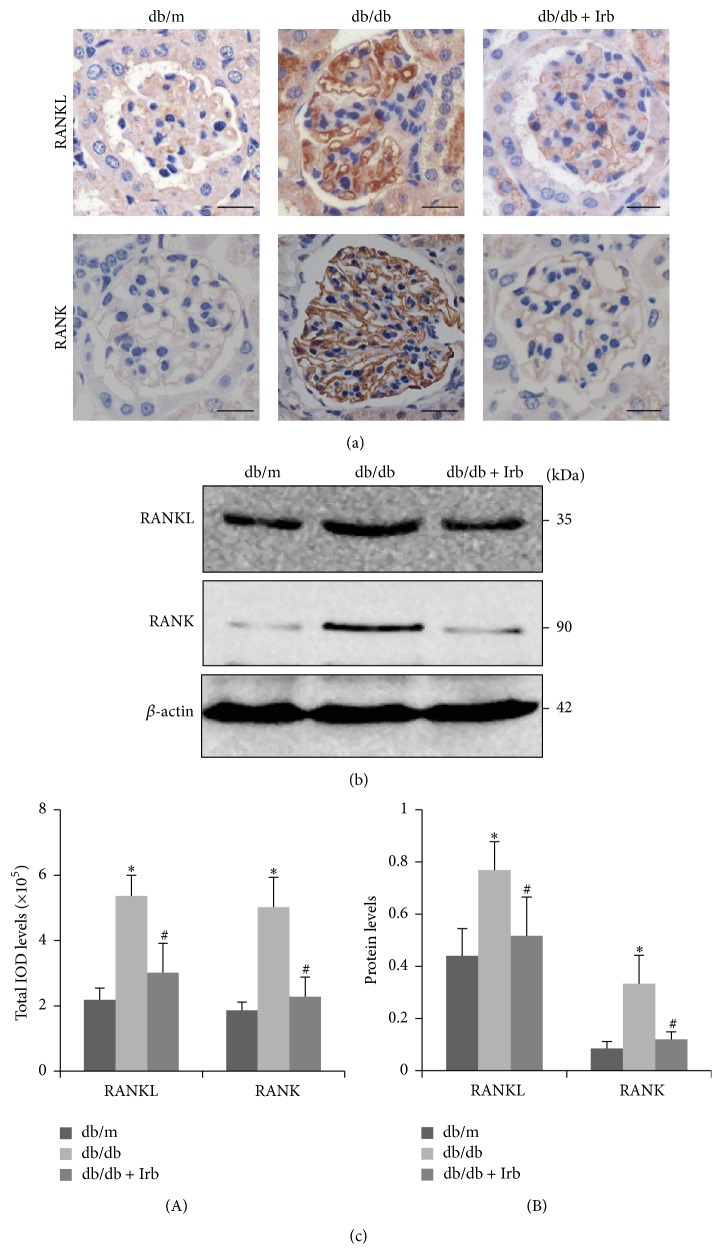
RANKL and RANK were overexpressed in db/db mice and downregulated by Irb. (a) Representative fields of RANKL and RANK, as labeled by immunohistochemical staining (scale bars: 50 *μ*m). (b) Representative immunoblots of RANKL and RANK expression in the kidney. (c) Quantification of the immunohistochemical staining (A). Quantification of the immunoblot (B): the ratio between RANKL and *β*-actin and the ratio between RANK and *β*-actin are presented. The bars in panel (c) show the mean expression in arbitrary units (error bars, S.D.). ^*∗*^ *P* < 0.05 compared with db/m; ^#^ *P* < 0.05 compared with db/db, *t*-test.

We investigated whether RANKL and RANK were overexpressed in db/db mice. As shown in Figure 5, db/db mice had a significantly increased level of immunohistochemical staining for RANKL and RANK compared with db/m mice (Figures 5(a) and 5(c)(A)). We found that RANKL and RANK were mainly expressed in the glomeruli, along the glomerular capillary loop in db/db mice. Moreover, the protein levels of RANKL and RANK were also significantly higher in the kidneys of db/db mice (Figures 5(b) and 5(c)(B)). After 12 weeks of treatment with Irb, RANKL and RANK were downregulated (Figures 5(a)–5(c)).

### 3.7. Irb Inhibited NF-*κ*B Pathway Activation in db/db Mice

**Figure 6:**
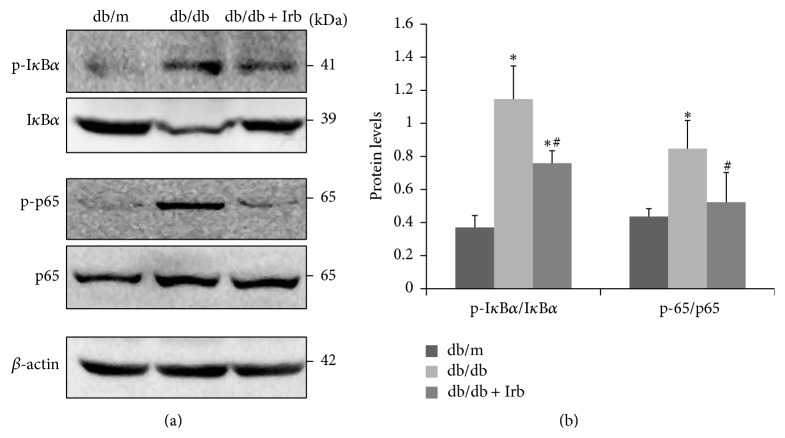
Irb inhibited NF-*κ*B pathway activation in db/db mice. (a) Representative immunoblot of p-I*κ*B*α*, I*κ*B*α*, p-p65, and p65 in the kidney. (b) Quantification of the immunoblot: the ratio between p-I*κ*B*α* and I*κ*B*α* and the ratio between p-p65 and p65 are presented. The bars in panel (b) show the mean expression in arbitrary units (error bars, S.D.). ^*∗*^ *P* < 0.05 compared with db/m; ^#^ *P* < 0.05 compared with db/db, *t*-test.

Compared with db/m mice, db/db mice showed significantly increased phosphorylation of I*κ*B*α* and NF-*κ*B/p65 and reduced I*κ*B*α* protein levels (Figures 6(a) and 6(b)), suggesting that the NF-*κ*B pathway is activated. After 12 weeks of treatment with Irb, NF-*κ*B activation in the db/db mice was blocked (Figures 6(a) and 6(b)).

## 4. Discussion

DN is a critical complication and a leading cause of ESRD in diabetes patients. Thus, a comprehensive understanding of the mechanisms underlying the pathogenesis of DN is urgently needed. Our present study investigated the role of RANKL and RANK in type 2 DN and whether the beneficial effects of Irb in DN are mediated by the RANKL-RANK-NF-*κ*B pathway. The major findings of our study are that (1) RANKL and RANK are overexpressed in kidneys of mice with type 2 DN; (2) the NF-*κ*B pathway is activated in DN; (3) Irb treatment alleviates diabetes-induced renal dysfunction, podocyte injury, and MCP-1 expression; and (4) the beneficial effects of Irb in DN are associated with its ability to suppress RANKL/RANK and the downstream NF-*κ*B pathway. To the best of our knowledge, our study indicates, for the first time, that RANKL and RANK play important roles in the development of DN and that they are key targets of Irb treatment.

T2DM, the more prevalent form of diabetes in diabetic patients, is characterized by hyperglycemia, hyperlipidemia, and insulin resistance [25]. In our study, we used db/db mice, which are a spontaneous T2DM mouse model, to explore the development and treatment of DN. At 8 weeks of age, db/db mice showed increased ACR, suggesting the occurrence of DN. Then, we started to treat one group of the db/db mice with Irb for 12 weeks. At the end of the experiment, the db/db mice showed severe metabolic abnormalities, including elevated FBG, FINS, TG, TC, and BW. Additionally, as evaluated by renal function and histopathology analysis, our db/db mice revealed overt DN, showing increased BUM, Scr, and ACR accompanied by mesangial expansion, podocyte foot process effacement, and thickening of the GBM.

In diabetes, the renin-angiotensin system (RAS) of the kidneys is activated. The RAS plays an important role in the regulation of systemic blood pressure. Angiotensin II, the final product of this system, which constricts blood vessels and raises blood pressure, mainly binds to angiotensin II type 1 receptor (AT1-R) and AT2-R [26]. Because the major pathogenic signaling of angiotensin II is mediated by AT1-R, AT1-R blockers (ARBs) are widely used in patients with hypertension and cardiovascular diseases. Moreover, accumulating evidence has suggested that ARBs have beneficial effects in patients with DN [11–15, 27, 28]. According to the available data from clinical trials, Irb, a widely used ARB in clinical practice, has a more pronounced pharmacological effect than other compounds within the same class. Based on two studies from the New England Journal of Medicine [27, 28], Irb is effective in treating both early and late stage nephropathy in hypertensive patients with T2DM. Additionally, the Irbesartan Diabetic Nephropathy Trial conducted by Lewis revealed that Irb slows the progression of nephropathy in patients with type 2 diabetes independently of its blood pressure-lowering effect. In animal studies, Tunçdemir and Öztürk [29] found that Irb exerts its renoprotective effects, including regulating renal hemodynamics and controlling tissue damage, by preventing podocyte loss. Bonnet et al. [30] investigated the effect of Irb on streptozotocin-induced diabetic spontaneously hypertension rats. They demonstrated the efficacy of Irb in reducing albuminuria, which was associated with the downregulation of nephrin at the gene and protein level. Data from Hartner et al. [15] study showed that low dose Irb increases synaptopodin, which is a marker of functional podocytes, and reduces desmin, which is a marker of podocyte damage in renal tissue at the protein level. These changes occurred in response to a reduction in blood pressure and inflammation but not a reduction of ER stress and apoptosis. In the present study, we found that Irb treatment ameliorated podocyte foot process effacement, upregulated the expression of nephrin, and decreased the level of MCP-1 in both urine and renal tissue, suggesting that Irb may exert its major renoprotective effects by preventing podocyte injury and controlling inflammation in the kidney.

To gain further insights into the potential mechanism involved in the renoprotective effect of Irb in DN, we focused on RANKL and RANK. The available evidence implicates RANKL and RANK as mediators of inflammation in T2DM [20]. Mice treated with hydrodynamic injection of Rank shRNA lentiviral vectors show inactivation of NF-*κ*B and the inhibition of downstream proinflammatory signaling in the liver. However, Liu et al. [18] reported that RANK and RANKL comprise a novel receptor-ligand pair in the survival response of injured podocytes. In their study, they found that the knockdown of RANK alone did not induce podocyte apoptosis but mildly and nonsignificantly increased the apoptosis of podocytes exposed to PAN. However, the application of exogenous RANKL significantly reduced the apoptosis of podocytes transfected with or without RANK siRNA when exposed to PAN. Their results show that RANK plays an insignificant role in the protection of podocytes and that RANKL reduces podocyte apoptosis independently of RANK. Thus, whether RANKL acts on RANK to protect podocytes from apoptosis remains controversial. In our opinion, RANKL may exert its antiapoptotic effect on PAN-induced podocytes by binding the decoy receptor OPG, which acts as a survival factor for endothelial cells [17], pancreatic *β*-cells [31], and tubular cells [32, 33]. When binding to RANK, RANKL activates the NF-*κ*B pathway and subsequently invokes downstream proinflammatory signaling. Consistent with this pathway, our present results showed that RANKL and RANK were markedly increased in the kidneys of db/db mice, and the NF-*κ*B pathway and its downstream inflammatory mediator MCP-1 were subsequently activated. Irb treatment reduced the elevated RANKL and RANK and inhibited activation of the NF-*κ*B pathway.

In the present study, we demonstrated the renoprotective effects of irbesartan on DN. However, some limitations of our study are noted. First, the spontaneous T2DM db/db mouse model, which shares many metabolic phenotypes and renal abnormalities that are observed in patients, may still have some limited relevance to clinical conditions. Second, our study demonstrated the involvement of RANKL and RANK in the development of DN. However, the precise role of RANKL and RANK in diabetes-induced renal dysfunction and podocyte injury has not been definitively verified. Further, in vivo experiments employing RANK-specific inhibitors or RANK gene silencing are preferential to clarify the underlying mechanism.

## 5. Conclusions

In conclusion, Irb alleviated renal dysfunction, podocyte injury, and MCP-1 expression in DN. The molecular mechanism responsible for the protective effects of Irb may involve inhibition of the RANKL-RANK-NF-*κ*B pathway. Our study provides a novel mechanism through which Irb treats DN.

## Figures and Tables

**Table 1:** Animal characteristics at the end of the experiment.

	db/m	db/db	db/db + Irb
FBG (mmol/L)	6.15 ± 0.96	28.17 ± 3.70^*∗*^	25.51 ± 4.06^*∗*^
FINS (ng/mL)	1.53 ± 0.50	5.64 ± 1.12^*∗*^	5.14 ± 1.03^*∗*^
TC (mmol/L)	1.96 ± 0.27	5.21 ± 0.72^*∗*^	3.12 ± 0.93^#^
TG (mmol/L)	0.54 ± 0.06	2.55 ± 0.60^*∗*^	1.08 ± 0.61^#^
BW (gram)	25.85 ± 1.70	46.61 ± 3.31^*∗*^	44.56 ± 3.95^*∗*^

Data are the mean ± SD, *n* = 12 per group. ^*∗*^ *P* < 0.05 versus db/m. ^#^ *P* < 0.05 versus db/db.
