# Corrigendum to “Irbesartan Ameliorates Diabetic Nephropathy by Suppressing the RANKL-RANK-NF-*κ*B Pathway in Type 2 Diabetic db/db Mice”

**DOI:** 10.1155/2016/3151986

**Published:** 2016-10-18

**Authors:** Xiao-Wen Chen, Xiao-Yan Du, Yu-Xian Wang, Jian-Cheng Wang, Wen-Ting Liu, Wen-Jing Chen, Hong-Yu Li, Fen-Fen Peng, Zhao-Zhong Xu, Hong-Xin Niu, Hai-Bo Long

**Affiliations:** ^1^Department of Nephrology, ZhuJiang Hospital, Southern Medical University, Guangzhou 510280, China; ^2^Department of Gerontology, ZhuJiang Hospital, Southern Medical University, Guangzhou 510280, China; ^3^Department of Emergency, ZhuJiang Hospital, Southern Medical University, Guangzhou 510280, China

In the article titled “Irbesartan Ameliorates Diabetic Nephropathy by Suppressing the RANKL-RANK-NF-*κ*B Pathway in Type 2 Diabetic db/db Mice” [1], in Figure 3(c)(A), the value of ordinate should be narrowed down to 0.01 times that of the data presented. And in Figure 6(b), the abscissa p-65/p65 should be changed to p-p65/p65. The two corrected figures are presented here.

## Figures and Tables

**Figure 3:**
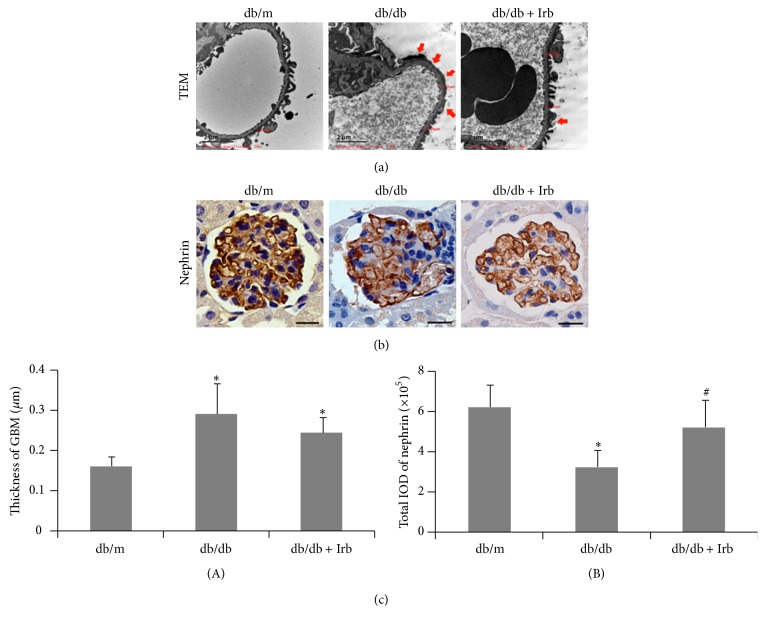
Irb alleviated diabetes-induced podocyte injury and the thickening of the GBM. (a) Representative fields of podocyte foot processes under TEM (scale bars: 2 *μ*m; red arrow indicates podocyte foot process effacement). (b) Representative fields of nephrin, as labeled by immunohistochemical staining (scale bars: 50 *μ*m). (c) Quantification of the GBM thickness (A) and immunohistochemical staining (B). The bars in panel (c) show the mean expression in arbitrary units (error bars, SD). ^*∗*^ *P* < 0.05 compared with db/m; ^#^ *P* < 0.05 compared with db/db, *t*-test.

**Figure 6:**
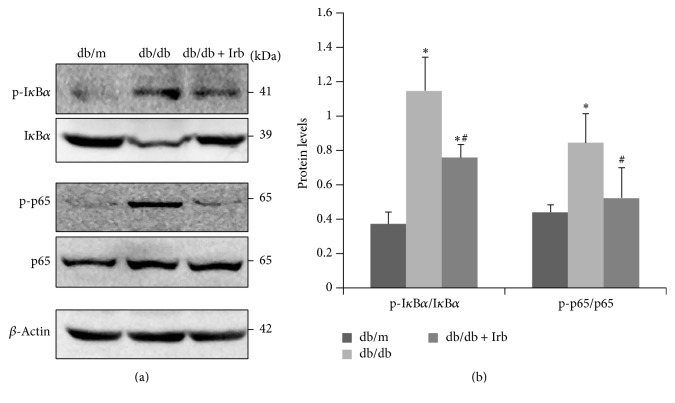
Irb inhibited NF-*κ*B pathway activation in db/db mice. (a) Representative immunoblot of p-I*κ*B*α*, I*κ*B*α*, p-p65, and p65 in the kidney. (b) Quantification of the immunoblot: the ratio between p-I*κ*B*α* and I*κ*B*α* and the ratio between p-p65 and p65 are presented. The bars in panel (b) show the mean expression in arbitrary units (error bars, SD). ^*∗*^ *P* < 0.05 compared with db/m; ^#^ *P* < 0.05 compared with db/db, *t*-test.
